# Defining the habitat niche of *Alopecurus myosuroides* at the field scale

**DOI:** 10.1111/wre.12300

**Published:** 2018-03-23

**Authors:** H Metcalfe, A E Milne, R Webster, R M Lark, A J Murdoch, L Kanelo, J Storkey

**Affiliations:** ^1^ Rothamsted Research Harpenden UK; ^2^ School of Agriculture, Policy and Development University of Reading Reading UK; ^3^ School of Biosciences University of Nottingham Sutton Bonington UK; ^4^ Sheffield UK

**Keywords:** weed patches, black‐grass, soil, habitat, precision agriculture

## Abstract

The distribution of *Alopecurus myosuroides* (black‐grass) in fields is patchy. The locations of these patches can be influenced by the environment. This presents an opportunity for precision management through patch spraying. We surveyed five fields on various types of soil using a nested sampling design and recorded both *A. myosuroides* seedlings in autumn and seed heads in summer. We also measured soil properties at those sampling locations. We found that the patches of seed heads within a field were smaller than the seedling patches, suggesting that techniques for patch spraying based on maps of heads in the previous season could be inherently risky. We also found that the location of *A. myosuroides* patches within fields can be predicted through their relationship with environmental properties and that these relations are consistent across fields on different soil types. This improved understanding of the relations between soil properties and *A. myosuroides* seedlings could allow farmers to use pre‐existing or suitably supplemented soil maps already in use for the precision application of fertilisers as a starting point in the creation of herbicide application maps.

## Introduction

*Alopecurus myosuroides* Huds. (black‐grass) is one of the most common grass weeds of winter cereals in north‐west Europe (Holm *et al*., 1997) and is particularly problematic in the UK. *Alopecurus myosuroides* has a high reproductive rate and competes strongly with the cereal crops (Maréchal & Henriet, 2012). When mature, *A. myosuroides* plants produce large amounts of seeds, and so small failures in control can lead to rapid population growth and dense infestations. For many farmers, the main option for control of *A. myosuroides* and other weeds in the UK is the application of herbicides. These are often the sole method of control. In 2015, in the UK, 4 241 507 kg of herbicides were applied to cereal crops (Fera Science Ltd, 2017). Many farmers apply herbicides uniformly across individual fields and use on average six herbicidal active substances in a season for an arable crop (Garthwaite *et al*., 2014). Despite this heavy reliance on multiple chemical controls, many farmers are experiencing waning effectiveness owing to the evolution of herbicide resistance (Heap, 2017). These farmers are seeking alternative methods of weed management.

In addition to the need to delay or avoid the evolution of herbicide resistance, there are two further reasons to reduce herbicide use. First, agrochemicals can have negative impacts on the environment. Their inappropriate use can lead to contamination of surface water, ground water and the atmosphere (Garibay *et al*., 2001); this may contribute to loss of biodiversity, loss of ecosystem function and contamination of drinking water. Second, an increasing number of regulations are being placed on herbicides, and so, by reducing their use, farmers would become less reliant on individual active ingredients that could be withdrawn in future. The benefits of minimising herbicide use are therefore multiple: selection pressure would be reduced, the effective life of some active ingredients would be prolonged, environmental concerns would be reduced, and there would be less reliance on this single method of control, thereby encouraging greater adoption of integrated weed management programmes. One opportunity for reducing herbicide inputs is to spray only those areas of the field where weeds are a problem (site‐specific weed management).

*Alopecurus myosuroides*, like many weed species, grows in patches within fields. These patches can vary in size and shape (Cardina *et al*., 1997; Dieleman *et al*., 2000; Walter *et al*., 2002; Heijting *et al*., 2007). Nevertheless, these patches can be fairly stable, with core areas of *A. myosuroides* patches moving only 3–4 m over several years (Lutman *et al*., 2002). Patchiness can lead to many inefficiencies in weed management, as often farmers spray whole fields if average weed densities exceed some economic threshold related to profitability. However, there may be large parts of their fields that do not require spraying. Blanket spraying wastes time, energy and chemical (Cardina *et al*., 1997). Advances in global positioning technology and precision sprayers now make it possible to manage weeds at a much finer spatial resolution than was previously possible. There are two methods through which such forms of patch management can be achieved (Walter *et al*., 2002). The first is an offline system using treatment maps. These can be created from manually sampled data on weed distributions. Some of these maps are of inadequate quality, often because the sampling on which they are based was too sparse (Metcalfe *et al*., 2016). The second online approach is through real‐time detection of weeds with optical sensors, usually detecting mature weeds in the previous cropping season to guide spraying decisions in the following year. This approach is still in development, and while already feasible, it is not yet at the stage of widespread commercialisation (e.g. Murdoch *et al*., 2010, 2014).

Despite the numerous benefits of patch spraying as a form of weed management, it is not being taken up as a standard management tool. There may be several reasons for this (Christensen *et al*., 2009), perhaps the most difficult to counter being the inherent conservativeness of farmers when it comes to weed control. Given the consequences of a control failure, the concept of leaving some areas of the field unsprayed is currently seen as an unacceptable risk.

There is some indication that the patchy distribution of *A. myosuroides* is related to the similar variation in the soil (Holm, 1997; Lutman *et al*., 2002; Murdoch *et al*., 2014). Our lack of understanding of what determines the field‐scale habitat niche of this important species is currently preventing the implementation of site‐specific management. Understanding where weeds are in a field and what is determining their spatial distribution might not only reduce input costs, but also lead to the more accurate application of other control practices where needed (Dieleman *et al*., 2000), including variable seed rates and fertiliser applications. If we can understand how patches relate to soil, we might explain the observed distribution on *A. myosuroides* in each field but also define the potential habitat into which it could spread. In so doing, we could build insurance into any patch spraying protocol. This would also allow the use of existing or supplemented soil maps.

Previous investigators who have attempted to link *A. myosuroides* density and soil properties have limited their scope, sampling only at a single scale (e.g. Dunker & Nordmeyer, 1999, 2000; Lutman *et al*., 2002). This has led to conflicting results from different studies. Metcalfe *et al*. (2016) proposed a solution to resolve discrepancies in field studies. They found that relations that occur at certain scales could be obscured by uncorrelated variations at other scales, if only the overall correlation were calculated from all the data from a simple random sample. They successfully demonstrated in one field that relations between *A. myosuroides* and soil properties depend on the spatial scale and that different results can be obtained from different sampling scales. We applied this approach to five winter wheat fields with contrasting soil types over several seasons to investigate the relationships between soil properties and both *A. myosuroides* seedling counts and seed head counts. We set out to test three hypotheses:

1. The counts of seedlings and heads at each sampling location are similar, which if true means, patch spraying can be based on head counts recorded in the previous growing season,
2. Variance within fields of the distribution of *A. myosuroides* depends on relationships with soil properties at specific spatial scales, and
3. These relationships are similar from field to field.

By addressing these hypotheses, we tested whether farmers could use soil maps in the management of *A. myosuroides* and whether the scale of these relationships is appropriate for precise management of the weed.

## Materials and methods

### Field sites

We chose five sites with a range of soil types. Each site consisted of one field, which was in commercial winter wheat production in the season of study. All fields were in the South East of England (the main centre of *A. myosuroides* distribution) and reported by the farmers to have patchy *A. myosuroides* populations. The fields were separated by a minimum distance of 5.3 km and maximum of 65.6 km. Here, we refer to the fields by their location in Radbrook (Berkshire), Harpenden (Hertfordshire), Redbourn (Hertfordshire), Ivinghoe (Buckinghamshire) and Haversham (Buckinghamshire). Radbrook was studied in the 2012–2013 season, Harpenden in the 2013–2014 season, Redbourn and Ivinghoe in the 2014–2015 season, and Haversham in the 2015–2016 season.

### Nested sampling

**Figure 1:**
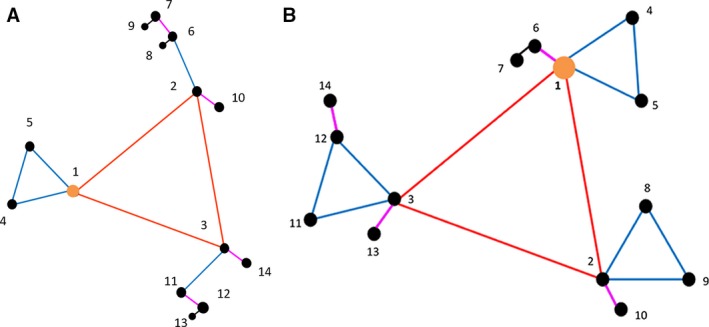
Nested sampling designs used (A) Harpenden and Radbrook and (B) Redbourn, Ivinghoe and Haversham. Vertices are labelled as the numbers 1–14. The yellow disc indicates the main station of the motif. Designs were separated by distances from level one. Red lines represent nodes spaced at level two of the design, blue lines indicate level three, purple lines link points at level four, and black lines represent level five.

We used an unbalanced nested sampling scheme as described by Metcalfe *et al*. (2016). The design was organised hierarchically with five levels. Each level corresponded with a specific scale of study, with level 1 defining the coarsest scale in each study and level 5 the finest (Fig. 1). The level 1 variation is represented by differences between the groups of sample sites associated with each main station in each field. Note that while the distances between points were constrained by the design, the directions were randomised independently in each main station. We sampled at nine such clusters in each field. Sampling sites were nested hierarchically in groups associated with each main station per the distances indicated in Table 1. We used an initial design with five scales (detailed in Table 1) in the first two fields at Radbrook and Harpenden. Based on the results from these two fields, we optimised the design, as described by Metcalfe *et al*. (2016), for use in the other three fields. This optimised design used coarser scales (Table 1) to try to capture better some of the coarse‐scale variation in *A. myosuroides* observed in the first two fields. To map the distribution of A.* myosuroides* and associated soil properties by kriging, we added 10 more sampling points in each field to fill the larger gaps in the coverage and thereby diminish the errors in prediction.

**Table 1:** Scales used at each level of the nested sampling design in each field. The nested design consists of five levels as described by Metcalfe *et al*. (2016). Level one represents the coarsest scales, and with each subsequent level, the scale is made finer. The design was refined after the first year's results from Harpenden and Radbrook, explaining the difference in the scales from the remaining study fields

Level of nested sampling design	Scale (m)				
	Harpenden	Radbrook	Redbourn	Ivinghoe	Haversham
1	50+	50+	60+	60+	60+
2	20	20	40	40	40
3	7.3	7.3	11.5	11.5	11.5
4	2.7	2.7	3.4	3.4	3.4
5	1	1	1	1	1

We located the positions for each main station at level 1 of the design by GPS (Topcon/Trimble, 2 cm accuracy). Each subsidiary sampling point was located by its distance and orientation from the main station by tape measure and compass. To define the sample support, we placed square quadrats (0.5 m^2^) on the ground with their south‐west vertices at the sampling point.

### Weed counts

We counted *A. myosuroides* seedlings within each quadrat in late autumn, while the plants were at the one‐ to two‐leaf stage. For fields where pre‐emergence herbicides were to be applied by the farmer, we placed plastic sheets over the sample quadrats for up to 24 h over the period of spraying to prevent herbicide reaching the sampling area. Seedling counts were obtained at Harpenden, Redbourn, Ivinghoe and Haversham, but were not obtained at Radbrook as the field was included in the study too late for seedlings to be assessed.

We counted *A. myosuroides* heads within the month prior to harvest of the wheat crop. We included in the count any heads within the vertical area directly above the quadrat. We disregarded any heads falling outside the quadrat irrespective of whether the plant originated inside the quadrat. Head counts were obtained at Harpenden, Radbrook, Redbourn and Ivinghoe. Because of very dense *A. myosuroides* at Haversham, extensive lodging of the crop made head counts inaccurate.

### Soil analyses

We sampled the soil in early winter, following prolonged rainfall, when we presumed soil moisture to be at field capacity. We took two soil cores from each quadrat with a half‐cylindrical auger of diameter 3 cm to a depth of 28 cm. We measured the gravimetric water content in layers 0−10 cm and 10−28 cm by loss on oven‐drying at 105°C for all sites except Radbrook. At Radbrook, we calculated a measure of volumetric water instead from theta probe measurements of the soil surface layers. Other variables were analysed by a commercial soil testing company, SOYL (Newbury, UK), on samples pooled from the two cores within each quadrat. Organic matter was measured by loss on ignition. Available phosphorus (P) was measured in a sodium bicarbonate extract at pH 8.2. The pH was measured in water, and soil texture (particle‐size distribution) was determined by laser diffraction. We did not measure organic matter and available phosphorus at Radbrook.

### Topography

Elevation data (LIDAR) were downloaded from data.gov.uk for each field (except Ivinghoe where the data were unavailable) at a 1 m resolution. We converted these into aspect and slope information using ArcGIS spatial analyst. To include these as one variate in our analyses, we computed the solar energy received throughout 1 year following methods outlined by Frank and Lee (1966). This variable gave an indication of the susceptibility of different areas of the field to drying and drought stress.

### Analysis

We calculated summary statistics and Pearson's correlation coefficients for all data. Note, however, that our use of the nested sampling design does not lead to an unbiased estimate of the correlation, because it ignores the dependency structure imposed by the sampling. The first level of the analysis was performed at the level of individual fields (variograms and kriging, principal components analysis and nested analysis). We then tested the hypothesis that these relationships were consistent across fields using all the data in a combined model (regression analysis).

#### Variograms and kriging

To create maps of seedling densities, we estimated and modelled variograms from all data points from both the sampling design and the 10 additional points to quantify the spatial structure in the variance of the measured variables. We did this using GenStat (Payne, 2013). We used ordinary kriging to predict the variables of interest across the field at points on a 1 m grid and then contoured the predictions in ArcMap (ESRI) to generate maps.

#### Principal components analysis

To obtain an overall appreciation of the correlations among the soil properties and how the *A. myosuroides* counts fit into that structure, we did principal components analyses as follows. We standardised the soil variables to zero mean and unit variance and effectively did the analysis on the correlation matrix, **R**, for each field separately. We then computed the Pearson correlation coefficients between the component scores as

(1) $$b_{\mathrm{ij}}=a_{\mathrm{ij}}\sqrt{\frac{\lambda_{j}}{\sigma_{i}^{2}}}$$

where *a*_*ij*_ denotes the *i*th element of the *j*th eigenvector and *λ*_*j*_ is the *j*th eigenvalue of matrix **R**, and $\sigma_{i}^{2}$ is the variance of the *i*th original soil variable. We plotted the coefficients *b* for the two leading components in unit circles and then added to the graphs the correlation coefficients between the *A. myosuroides* counts, sometimes regarded as ‘passive variables’, and the two leading components as described by Abdi and Williams (2010).

#### Nested analysis

The nested design structure allows the partitioning of the components of variance for both *A. myosuroides* and soil properties at each of the spatial scales studied. We did this using the residual maximum likelihood (REML) estimator as described by Metcalfe *et al*. (2016). Following partitioning of the components of variance at the different spatial scales, we estimated the correlations between *A. myosuroides* and the soil properties at each scale where the estimated components of variance were positive. We calculated confidence intervals (95%) for the correlations by Fisher's *z*‐transform, with degrees of freedom appropriate to the number of sampled pairs at the corresponding level of the design. Where the confidence intervals excluded zero, we determined the correlation to be statistically significantly different from zero.

#### Regression analysis

We tested the hypothesis that the relationships between the variance in *A. myosuroides* density and soil properties quantified at the individual field scale were consistent across the five fields. In this type of analysis, it is important that all terms are independent. As our three soil texture variables (sand, silt and clay) sum to 100%, they cannot be independent. We used the additive log‐ratio transform to create two independent variables (the log of the ratio of silt to sand and the log of the ratio of clay to sand; Aitchison, 1986). We also removed the soil moisture content below 10 cm from this analysis, as it was strongly correlated with surface soil moisture content, which is more likely to be recorded in soil surveys.

We did a regression analysis using REML where the field was included as a random term. We included all environmental properties as main effects. For this analysis, we considered only the first‐order model for soil properties to retain sufficient degrees of freedom for the analysis. Terms were selected using backward elimination according to the largest *P*‐value given by an *F* test when that term was dropped. The best model was chosen when all remaining terms gave significant values (*P *=* *0.05) for an *F* test when dropped from the model.

We also looked at incorporating the spatial autocorrelation in *A. myosuroides* numbers into this regression analysis by including the field location and variogram parameters as random effects. Again, terms were selected using backward elimination according to the largest *P*‐value given by an *F* test when that term was dropped. We also considered the possibility of using maximum likelihood in the place of REML, as this method allows us to compare AIC values across models with different fixed effects. For this model, backward elimination was also used for term selection.

## Results

*Alopecurus myosuroides* was present in all five fields. Numbers of *A. myosuroides* seedlings were greatest in Haversham and least in Radbrook (Table 2). The fields spanned a range of soil types, and the soil properties we measured varied substantially from one field to another. There were also different levels of within‐field variation in soil properties (Table 2). For example, pH was highest in Ivinghoe and lowest in Radbrook, but Redbourn showed the greatest variation.

**Table 2:** Summary statistics for *Alopecurus myosuroides* counts and soil properties measured in each field

Variate	Mean	Minimum	Maximum	Standard deviation	Skewness
Harpenden					
*A. myosuroides* seedling counts (per 0.5 m^2^ quadrat)	28.8	0	326	51.0	3.022
*A. myosuroides* head counts (per 0.5 m^2^ quadrat)	18.6	0	266	48.4	3.361
Gravimetric water content in top 10 cm (%)	25.63	21.8	30.0	1.86	0.5796
Gravimetric water content 10–28 cm depth (%)	23.83	19.3	31.0	2.19	0.5529
Organic matter (%wet weight)	4.53	3.0	6.0	0.65	0.4515
Available phosphorus (mg L^−1^)	24.70	11.0	54.4	8.30	1.2711
pH	6.90	6.1	7.8	0.28	0.2452
Sand (% wet weight)	32.1	17	51	4.9	0.413
Silt (% wet weight)	39.5	25	50	4.3	0.079
Clay (% wet weight)	28.4	23	39	3.0	0.846
Radbrook					
*A. myosuroides* seedling counts (per 0.5 m^2^ quadrat)	a	a	a	a	a
*A. myosuroides* head counts (per 0.5 m^2^ quadrat)	4.2	0	95	14.3	4.250
Volumetric water content in top 10 cm (%)	18.02	12.6	27.1	2.30	0.4134
Gravimetric water content 10–28 cm depth (%)	a	a	a	a	a
Organic matter (%wet weight)	a	a	a	a	a
Available phosphorus (mg L^−1^)	a	a	a	a	a
pH	5.87	4.9	6.9	0.45	0.1530
Sand (% wet weight)	33.5	15	53	7.9	0.137
Silt (% wet weight)	60.1	44	75	6.2	−0.078
Clay (% wet weight)	6.4	3	12	2.1	0.306
Redbourn					
*A. myosuroides* seedling counts (per 0.5 m^2^ quadrat)	12.8	0	129	20.4	2.658
*A. myosuroides* head counts (per 0.5 m^2^ quadrat)	11.0	0	107	21.3	2.623
Gravimetric water content in top 10 cm (%)	20.63	16.3	25.2	1.71	0.2640
Gravimetric water content 10–28 cm depth (%)	20.80	16.8	25.0	1.96	0.3887
Organic matter (%wet weight)	4.67	3.4	6.9	0.73	0.6735
Available phosphorus (mg L^−1^)	25.93	12.6	44.6	6.85	0.4422
pH	7.09	5.6	8.3	0.65	−0.1315
Sand (% wet weight)	28.4	9	46	5.5	0.175
Silt (% wet weight)	44.3	34	68	5.0	1.053
Clay (% wet weight)	27.3	15	38	4.2	0.537
Ivinghoe					
*A. myosuroides* seedling counts (per 0.5 m^2^ quadrat)	3.3	0	84	10.2	5.929
*A. myosuroides* head counts (per 0.5 m^2^ quadrat)	6.1	0	172	22.5	5.817
Gravimetric water content in top 10 cm (%)	22.34	18.7	24.8	0.91	−0.6583
Gravimetric water content 10–28 cm depth (%)	21.06	18.2	23.9	1.07	−0.0209
Organic matter (%wet weight)	4.73	3.6	5.7	0.43	0.0294
Available phosphorus (mg L^−1^)	14.29	9.6	23.4	2.58	0.6174
pH	8.11	7.7	8.5	0.14	0.0927
Sand (% wet weight)	22.1	11	47	8.2	1.335
Silt (% wet weight)	28.8	11	38	4.2	−0.720
Clay (% wet weight)	49.1	33	63	5.7	−0.632
Haversham					
*A. myosuroides* seedling counts (per 0.5 m^2^ quadrat)	63.6	0	488	111.9	2.030
*A. myosuroides* head counts (per 0.5 m^2^ quadrat)	a	a	a	a	a
Gravimetric water content in top 10 cm (%)	22.49	17.4	28.2	2.13	0.3929
Gravimetric water content 10–28 cm depth (%)	20.92	15.9	26.0	1.93	0.1560
Organic matter (%wet weight)	4.26	3.1	5.8	0.53	0.3124
Available phosphorus (mg L^−1^)	9.07	4.8	16.0	2.43	0.7981
pH	7.21	6.5	7.9	0.29	−0.3882
Sand (% wet weight)	44.9	23	62	8.6	−0.508
Silt (% wet weight)	29.6	22	38	3.7	−0.039
Clay (% wet weight)	25.5	16	40	5.4	0.9525

a Missing data.

The relationships between *A. myosuroides* and soil properties as expressed by Pearson's correlations were strong for water, organic matter and texture (Table 3). Other soil properties, such as available phosphorus, were only weakly correlated with *A. myosuroides* (Table 3). The relationships between *A. myosuroides* seedling counts and soil properties were stronger and more consistent across fields than between soil properties and head counts.

**Table 3:** Pearson's correlation coefficients between *Alopecurus myosuroides* seedling and head counts and soil properties in each field

Soil property	Harpenden		Radbrook		Redbourn		Ivinghoe		Haversham	
	Seedlings	Heads	Seedlings	Heads	Seedlings	Heads	Seedlings	Heads	Seedlings	Heads
Gravimetric water content in top 10 cm (%)†	**0.482**	**0.279**	b	**0.292**	**0.321**	0.172	0.101	0.080	**0.616**	b
Gravimetric water content 10–28 cm depth (%)	**0.491**	**0.342**	b	b	**0.519**	**0.280**	−0.172	−0.051	**0.448**	b
Organic matter (%wet weight)	**0.527**	**0.309**	b	b	**0.462**	**0.269**	−0.080	0.108	**0.349**	b
Available phosphorus (mg L^−1^)	0.023	0.041	b	b	−0.132	−**0.184**	−0.132	−0.011	0.029	b
pH	−**0.475**	−**0.310**	b	**0.337**	0.017	−0.062	−0.001	−0.094	0.112	b
Sand (% wet weight)	0.135	0.139	b	−**0.189**	0.049	0.007	−**0.235**	−0.157	−**0.253**	b
Silt (% wet weight)	−**0.384**	−**0.264**	b	0.124	−**0.320**	−0.144	0.034	0.061	**0.176**	b
Clay (% wet weight)	**0.328**	0.152	b	**0.348**	**0.324**	0.165	**0.326**	**0.188**	**0.280**	b

This analysis takes all data into account, ignoring the nested sampling structure.

Two‐sided tests of correlations different from zero are marked in bold where significant (*P *≤* *0.05).

a Gravimetric water content was measured except for Radbrook where we measured volumetric water content.

b Missing data.

### Variograms and kriging

**Figure 2:**
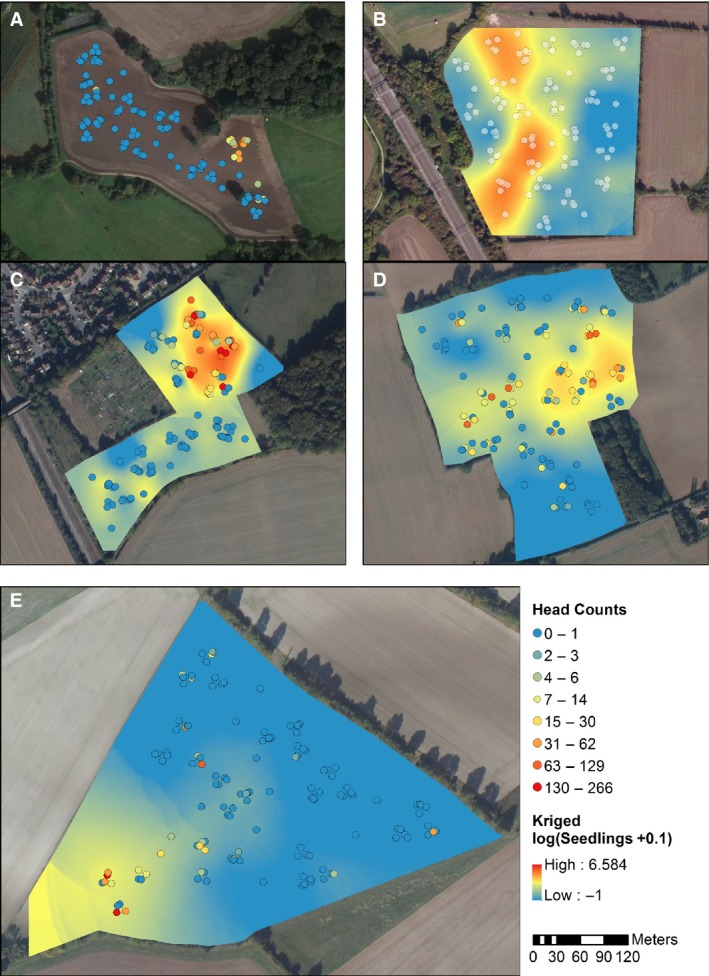
Maps showing the sampling locations (circles) in each of the five fields: (A) Radbrook (B) Haversham, (C) Harpenden, (D) Redbourn and (E) Ivinghoe. Where the circles are filled, the colour indicates the number of heads counted in a 0.5 m^2^ quadrat at that sampling location. Where the field is filled, the colour represents the kriged values for log (seedling counts +0.1) in a 0.5 m^2^ quadrat at each sampling location. The kriging was conducted using ordinary kriging based on the variogram fitted for that field.

Generally, the distribution of *A. myosuroides* heads within the fields showed the same pattern as for seedlings, but in many instances, the patches were smaller (Fig. 2). The distribution in all fields was patchy (Fig. 2) with all fields having some quadrats free of *A. myosuroides*.

In the kriged maps, there was some accord between *A. myosuroides* distribution (Fig. 2) and soil moisture (Figure S1), organic matter (Figure S2), clay content (Figure S3) and pH (Figure S4). It is also notable that at Radbrook and Ivinghoe, where the fewest *A. myosuroides* (Table 2) were, was also where we found the the driest soil and the most extreme values of soil pH (Figures S1 and S4).

### Principal components analysis

**Figure 3:**
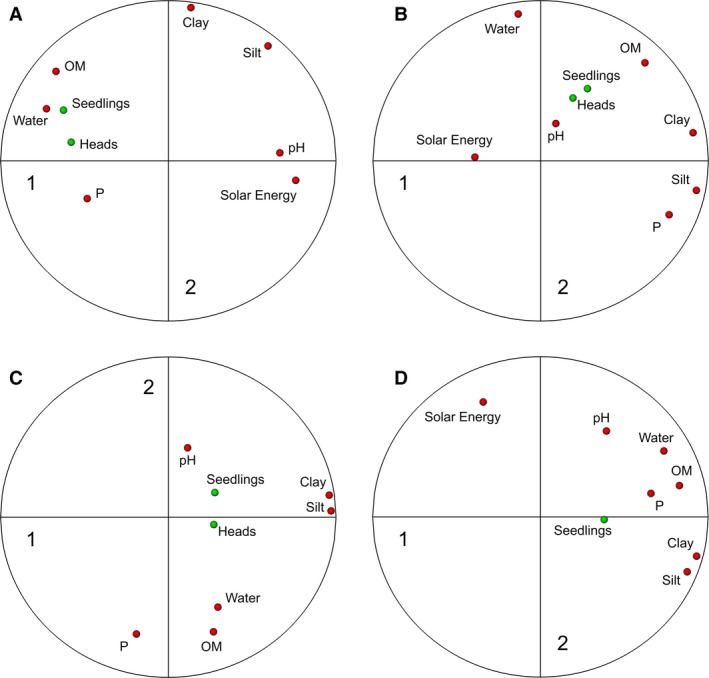
Principal component analysis on soil properties measured in each of the five study sites: (A) Harpenden, (B) Redbourn, (C) Ivinghoe, and (D) Haversham. The first two principal components are shown here with the loadings for each soil property shown with a solid arrow. The loadings for the *Alopecurus myosuroides* counts are projected onto the principal component plot (without being included in the analysis) to show how they relate to the soil properties. The length of the arrow shows the size of contribution to each principal component.

Within each field, we observed consistent covariation in soil properties (Fig. 3). The largest amount of variation (PC 1) in soil properties within a field was accounted for by soil texture and water. Soil pH explained an additional source of variation and generally corresponds with PC 2 (Fig. 3).

### Nested analysis

The scale‐dependent analysis of the nested design (Table 4) revealed much stronger correlations between *A. myosuroides* and particular soil properties than did the Pearson correlation. At medium to coarse scales, there were significant positive correlations between organic matter and the number of *A. myosuroides* seedlings in all fields except for Ivinghoe, which also had the least intrafield variance for this soil property. These relationships are particularly strong at coarse scales. Relationships were weaker for heads, and the only significant correlation between organic matter and heads was found in Harpenden at level 2 of the design. The patterns observed relating organic matter and *A. myosuroides* at Ivinghoe differ from the other four fields. In this field, the overall variation in organic matter was smaller than that in the other fields.

**Table 4:** Scale‐dependent correlations between various soil properties and *Alopecurus myosuroides* seedlings and heads

Scale	Harpenden		Radbrook		Redbourn		Ivinghoe		Haversham	
	Seedlings	Heads	Seedlings	Heads	Seedlings	Heads	Seedlings	Heads	Seedlings	Heads
Soil organic matter										
1	**0.99**	a	b	b	**0.69**	c	−0.08	0.21	**0.90**	b
2	0.01	−**0.62**	b	b	**0.68**	c	a	a	0.22	b
3	**0.39**	−0.05	b	b	**0.28**	c	−**0.32**	0.03	**0.62**	b
4	a	a	b	b	a	c	−**0.34**	−0.05	0.06	b
5	−0.05	−0.12	b	b	a	c	a	0.19	a	b
Soil water content in the top 10 cm (gravimetric water content was measured except for Radbrook where we measured volumetric water content)										
1	**0.93**	**0.91**	b	0.54	0.55	**0.92**	0.44	**0.73**	0.65	b
2	**0.57**	0.07	b	a	a	a	a	a	**0.71**	b
3	−**0.71**	**0.33**	b	a	a	a	a	a	**0.84**	b
4	a	a	b	a	a	0.32	−0.22	0.21	**0.99**	b
5	**0.93**	**0.91**	b	0.54	0.55	**0.92**	0.44	**0.73**	0.65	b
Soil pH										
1	−**0.89**	c	b	**0.80**	0.03	−0.32	−0.17	−**0.88**	c	b
2	−0.11	c	b	a	0.25	−0.02	a	a	c	b
3	−**0.49**	c	b	a	−0.21	a	a	a	c	b
4	a	c	b	−0.17	a	**0.79**	−**0.34**	a	c	b
5	0.22	c	b	−0.12	a	a	a	−0.36	c	b
Soil clay content										
1	**0.85**	**0.83**	b	0.61	**0.71**	c	0.45	0.44	0.55	b
2	0.28	0.05	b	a	0.32	c	a	a	0.22	b
3	**0.69**	0.25	b	**0.96**	**0.46**	c	a	a	**0.24**	b
4	a	a	b	**0.52**	−**0.88**	c	**0.36**	−0.06	0.08	b
5	−0.04	−0.18	b	−0.35	a	c	a	0.25	a	b

Correlation coefficients shown in bold are significantly different from zero (*P* ≤ 0.05).

a Indicates where a negative variance component was fitted using REML; as part of the nested analysis, these were found to be not significantly different from zero.

b Missing data.

c Indicates that no model could be fitted using REML.

Across all fields, there was a broad correspondence between *A. myosuroides* seedling and head numbers and moisture content (Table 4). This was confirmed by significant correlations at multiple scales for both seedlings and heads.

In Harpenden, we found a significantly strong negative correlation between *A. myosuroides* seedlings and pH at coarse and medium scales (Table 4). Ivinghoe, where the pH was similar, showed a significant negative relationship at the 3.4–11.5 m scale as well as a coarse‐scale negative relationship with *A. myosuroides* heads (Table 4). However, in Radbrook and Redbourn, where the soil is generally more acid, there were significant positive correlations (Table 4). These results suggest a nonlinear, unimodal relationship between pH and *A. myosuroides* and that a slightly acidic pH is the most favourable for *A. myosuroides*.

Soil texture is reported to be an important influence on the presence of *A. myosuroides* (Lutman, 2002), and our data supported this. There were significant positive correlations between clay and *A. myosuroides* at all sites with larger positive correlations tending to be at coarse scales (Table 4). The compositional nature of the relationship between the three texture variables means that we observed negative counterparts in silt and sand. We observed similar relationships emerging for heads, yet these tended to be much smaller correlation coefficients, indicating the link between soil texture and *A. myosuroides* was weaker for heads than was for seedlings (Table 4).

### Regression analysis

**Figure 4:**
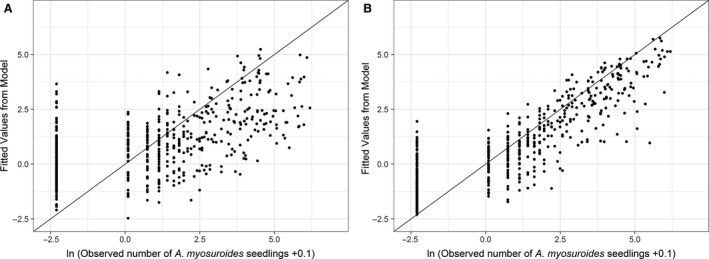
Scatter plots showing the relationship between the observed *Alopecurus myosuroides* seedling densities and the values predicted by the regression model. The non‐spatial model (A) incorporates the fixed effects as listed in Table 5 and field location as a random effect. The spatial model (B) also incorporates an estimation of the variogram to describe spatial autocorrelation in the *A. myosuroides* seedling counts.

When we considered all sites together as part of the regression analysis, a suite of soil properties including texture, water and topography (as defined by solar energy) (Table 5) provided a good prediction of *A. myosuroides* seedling densities (Fig. 4A). If we account for the autocorrelation in *A. myosuroides* seedling densities by fitting a spherical variogram with a nugget of 2.207, range 105.4 m and a sill of 1.298, then our predictive capability was further improved (Fig. 4B). Despite the autocorrelation giving us improved predictive power, there is still scope for soil properties to be used to improve the prediction with soil pH, water and topography significantly contributing to this model (Table 5). The same soil property terms were selected by the maximum likelihood approach, albeit with different effects due to the different type of model fitted. Where the fit of these models was poorest was when the observed data were zero. This demonstrates the inherent conservatism of this model, as where it is inaccurate, it will generally predict the presence of *A. myosuroides* when there is none.

**Table 5:** Terms selected in a regression‐type analysis using REML to predict *Alopecurus myosuroides* seedling densities from soil properties. The non‐spatial model has only field location as a random effect, whereas the spatial model allows the estimation of a variogram as a random effect. Here, a spherical variogram with a nugget of 2.207, range of 105.4 m and a sill of 1.298 was fitted

Term	Effect	SE
Non‐spatial model (AIC: 1305.51)		
Constant	0.9030	1.04080
Log(clay:sand)	2.131	0.6132
Log(silt:sand)	−1.524	0.6082
Gravimetric water content – top 10 cm	0.3806	0.06015
Solar energy	−0.002344	0.0004427
Spatial model (AIC: 1184.95)		
Constant	0.5675	0.62214
pH	0.6692	0.28583
Gravimetric water content – top 10 cm	0.2429	0.05839
Solar energy	−0.001669	0.0007076

**Figure 5:**
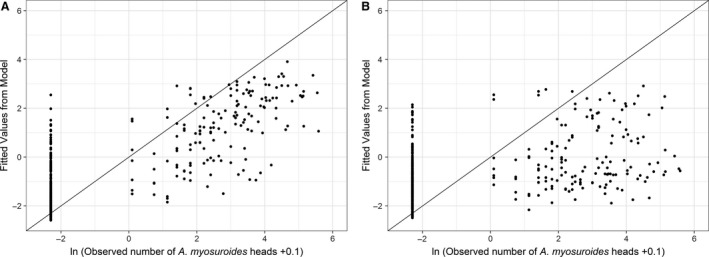
Scatter plots showing the relationship between the observed *Alopecurus myosuroides* head counts and the values predicted by the regression model. The non‐spatial model (A) incorporates the fixed effects as listed in Table S1 and field location as a random effect. The spatial model (B) also incorporates an estimation of the variogram to describe spatial autocorrelation in the *A. myosuroides* seedling counts.

Despite our ability to predict the density of *A. myosuroides* seedling populations from soil properties accurately, our experience for heads was less promising (Fig. 5). Again, the addition of information on the autocorrelation in head numbers (spherical model, nugget* *=* *2.470, range* *=* *122.3 m, sill* *=* *1.136) reduced the need for as many soil properties to be considered (Table S1). However, the predictive power was still poorer than for seedling densities (compare Fig. 5 with Fig. 4) and the model fitted using maximum likelihood incorporated different terms. The discrepancy between these two approaches indicates the lack of fit in these models and brings doubt as to the usefulness of using soil properties in the prediction of head densities.

## Discussion

Our results confirm that the distribution of *A. myosuroides* seedlings in the autumn can be patchy in fields growing winter wheat for commercial purposes (Fig. 2). We also found that the distribution of seed heads in the summer was a contraction of the initial *A. myosuroides* patch (Fig. 2). This observation is contrary to our first hypothesis and so highlights a problem associated with current methods of patch spraying, which map A*. myosuroides* heads in the summer to guide herbicide application of seedlings in the following season (Walter *et al*., 2002). If the contraction of patches is due to the environment, then this does not pose a risk to the farmer. However, if the contraction of patches during the growing season is due to effective management measures in the intervening period, then there is a risk that the patches could expand again if those same measures are not implemented in the following season.

Generally, there were strong correlations between *A. myosuroides* and soil properties that were associated with the first principal component of soil variation, namely soil texture, organic matter and water (Fig. 3, Table 4). These primary sources of variation could be linked to *A. myosuroides* seedling numbers by correlation at multiple spatial scales (Table 4) and so may be useful predictors of patch location. In addition, pH, a secondary source of within‐field variation in soil (Fig. 3), could also be linked to *A. myosuroides* seedling counts, and so measurement of this in the field is likely to provide more information than measurement of additional soil properties linked to the main source of variation (PC1 in Fig. 3).

When trying to predict *A. myosuroides* densities from soil properties, we found that the best predictors came from a regression model that considered the underlying autocorrelation in *A. myosuroides* seedling numbers (Fig. 4). In this model, information about soil improved that prediction, with soil moisture and pH being of importance (Table 5). These two soil properties represent the two main sources of variation in soil within the five fields (Fig. 3). Solar energy was also important, indicating that the topography of the fields is important for the distribution of *A. myosuroides* seedlings (Table 5). Areas of the fields with consistently dense *A. myosuroides* were characterised by large clay and organic matter content with a slightly acid pH and received little solar energy (meaning they were less prone to drying out).

Our findings were reasonably consistent across all five fields, which covered a few growing seasons and soil types. This provides some support for our third hypothesis and indicates that the patterns observed here may be general. The strongest relationships between soil properties and *A. myosuroides* we found were in Redbourn and Harpenden, the fields with intermediate infestation. Where infestation was greatest (Haversham) and particularly low (Ivinghoe and Radbrook), there were weaker correlations between *A. myosuroides* numbers and soil properties. This indicates that the relationship between *A. myosuroides* and soil properties might depend on plant density. Where *A. myosuroides* densities were low, the relationship with the soil was weak; the patch may not have reached all areas suitable for growth. Where densities are high, there might be spillover out of the optimal parts of the field; as seed production is so great, it is likely that some seed will germinate and the plants will grow even outside their optimal environment.

The use of soil properties in the prediction of patch locations looks promising as it is consistent across fields and seasons, especially if we consider the incorporation of spatial autocorrelation in the prediction of seedling numbers. Where our predictive power was poorest seems to be in the prediction of areas with no *A. myosuroides* seedlings (Fig. 4). However, our model is more likely to predict that there will be *A. myosuroides* present when there is none, making it low risk and so more likely to be useful to farmers.

The scale‐dependent correlations that provide the strongest links between *A. myosuroides* counts and soil properties were most often at coarse scales (Table 4). This is especially pertinent for weed management, as it is a scale that is useful for the farmer. Most machinery currently available on farm operates at scales of 20 m or greater and so it is helpful to know that this is a relevant scale for management, if patch spraying were to be implemented based on soil maps.

## Conclusions

Our results show that it is more important for farmers to be able to target patches of *A. myosuroides* seedlings than the mature plants, as the seedlings cover a greater part of the field. Seedling patches can be predicted by relationships with soil properties, and these relationships are consistent across fields. This improved understanding of the relationship between soil and *A. myosuroides* seedlings could allow pre‐existing, or supplemented soil maps already in use for the precision application of fertilisers, to be a useful starting point in the creation of herbicide application maps.

## Supporting information


**Table S1** Terms selected in a regression type analysis using REML to predict *A. myosuroides* head densities from soil properties.
**Figure S1** Maps showing the kriged soil moisture content (0–10 cm) in each of the 5 fields (a) Radbrook (b) Haversham, (c) Harpenden, (d) Redbourn, (e) Ivinghoe, soil moisture is gravimetric in all cases except Radbrook where the volumetric moisture content is shown.
**Figure S2** Maps showing the kriged soil organic matter measured by loss on ignition in each of the 5 fields (a) Harpenden (b) Redbourn, (c) Haversham, (d) Ivinghoe.
**Figure S3** Maps showing the kriged soil clay content in each of the 5 fields (a) Radbrook (b) Haversham, (c) Harpenden, (d) Redbourn, (e) Ivinghoe, soil moisture is gravimetric in all cases except Radbrook where the volumetric moisture content is shown.
**Figure S4** Maps showing the kriged soil pH in each of the 5 fields (a) Radbrook (b) Haversham, (c) Harpenden, (d) Redbourn, (e) Ivinghoe, soil moisture is gravimetric in all cases except Radbrook where the volumetric moisture content is shown.
