# Supplementary material for: Defining the habitat niche of Alopecurus myosuroides at the field scale
Source: Weed Res. 2018 Mar 23;58(3):165–76. doi: 10.1111/wre.12300 (PMC5993226; doi:10.1111/wre.12300)
Supplement: Supplementary file 1 — Table S1 Terms selected in a regression type analysis using REML to predict A. myosuroides head densities from soil properties. Figure S1 Maps showing the kriged soil moisture content (0–10 cm) in each of the 5 fields (a) Radbrook (b) Haversham, (c) Harpenden, (d) Redbourn, (e) Ivinghoe, soil moisture is gravimetric in all cases except Radbrook where the volumetric moisture content is shown. Figure S2 Maps showing the kriged soil organic matter measured by loss on ignition in each of the 5 fields (a) Harpenden (b) Redbourn, (c) Haversham, (d) Ivinghoe. Figure S3 Maps showing the kriged soil clay content in each of the 5 fields (a) Radbrook (b) Haversham, (c) Harpenden, (d) Redbourn, (e) Ivinghoe, soil moisture is gravimetric in all cases except Radbrook where the volumetric moisture content is shown. Figure S4 Maps showing the kriged soil pH in each of the 5 fields (a) Radbrook (b) Haversham, (c) Harpenden, (d) Redbourn, (e) Ivinghoe, soil moisture is gravimetric in all cases except Radbrook where the volumetric moisture content is shown. [file WRE-58-165-s001.docx]

**Supporting Information**

*Weed Research*

**Defining the habitat niche of black-grass (*Alopecurus myosuroides*) at the field scale.**

H METCALFE*†, A E MILNE*, R WEBSTER*, R M LARK‡, A J MURDOCH†, L KANELO† & J STORKEY*

*Rothamsted Research, Harpenden, Hertfordshire AL5 2JQ, UK, †School of Agriculture, Policy and Development, University of Reading, Earley Gate, PO Box 237, Reading RG6 6AR, UK, and ‡British Geological Survey, Keyworth, Nottingham NG12 5GG, UK

**Table S1** Terms selected in a regression type analysis using REML to predict A. myosuroides head densities from soil properties. The non-spatial model has only field location as a random effect, whereas the spatial model allows the estimation of a variogram as a random effect. Here a spherical variogram with a nugget of 2.470, range of 122.3m and a sill of 1.136 was fitted.

| **Term** | **Effect** | **S.E.** |
| --- | --- | --- |
| **Non-spatial model** | | |
| Constant | -0.8577 | 1.33253 |
| Log(clay:sand) | 2.292 | 0.4448 |
| Log(silt:sand) | -1.998 | 0.5245 |
| Soil Organic Matter | 0.7466 | 0.20514 |
| Gravimetric water content – top 10 cm | 0.3269 | 0.08080 |
| **Spatial model** |  |  |
| Constant | -1.023 | 0.3454 |
| Phosphorus | -0.0451 | 0.019264 |
| Gravimetric water content – top 10 cm | 0.1609 | 0.07105 |

**Fig. S1** Maps showing the kriged soil moisture content (0-10 cm) in each of the 5 fields a) Radbrook b) Haversham, c) Harpenden, d) Redbourn, e) Ivinghoe, soil moisture is gravimetric in all cases except Radbrook where the volumetric moisture content is shown. The kriging was conducted using ordinary kriging based on the variogram fitted for that field.

| 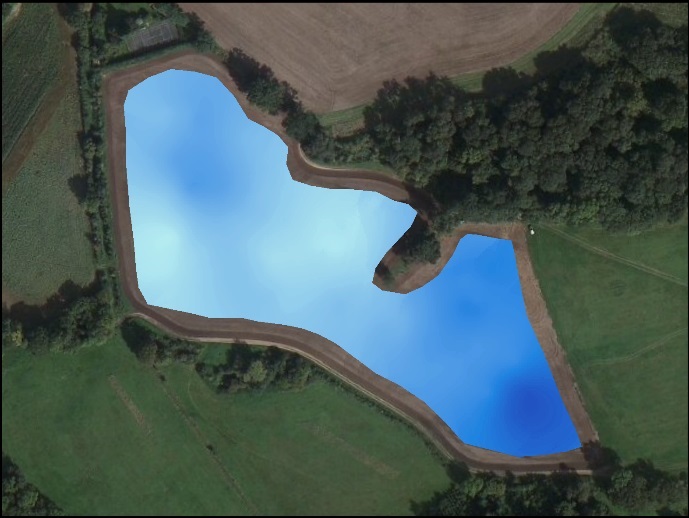  a | 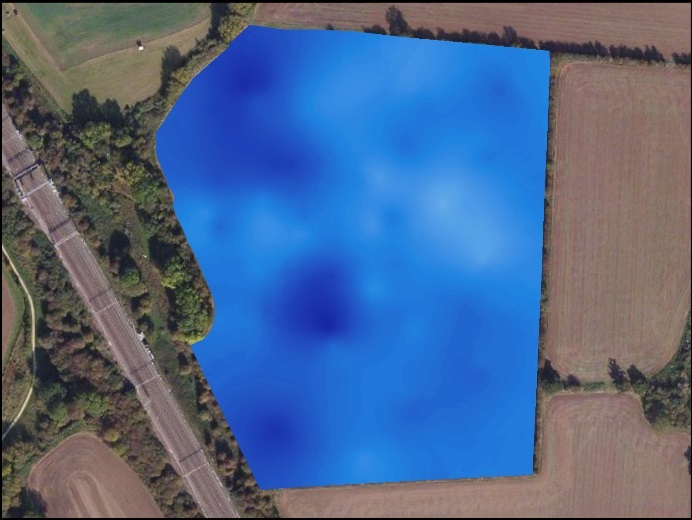  b |
| --- | --- |
| 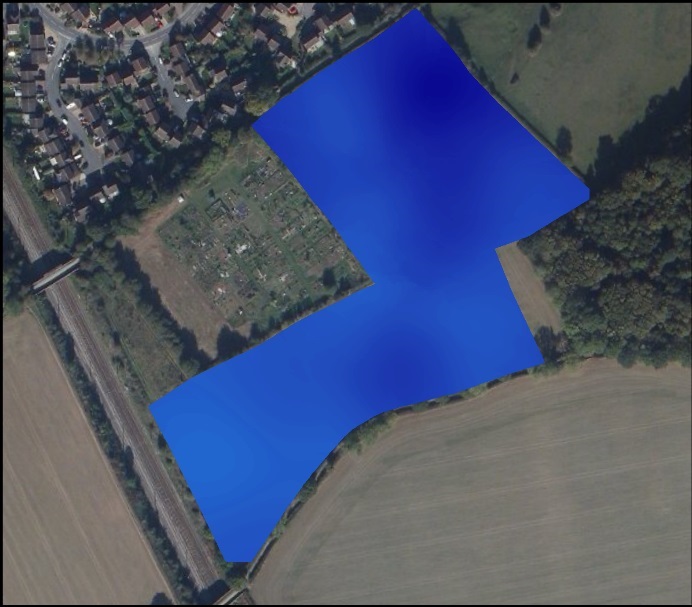  c | 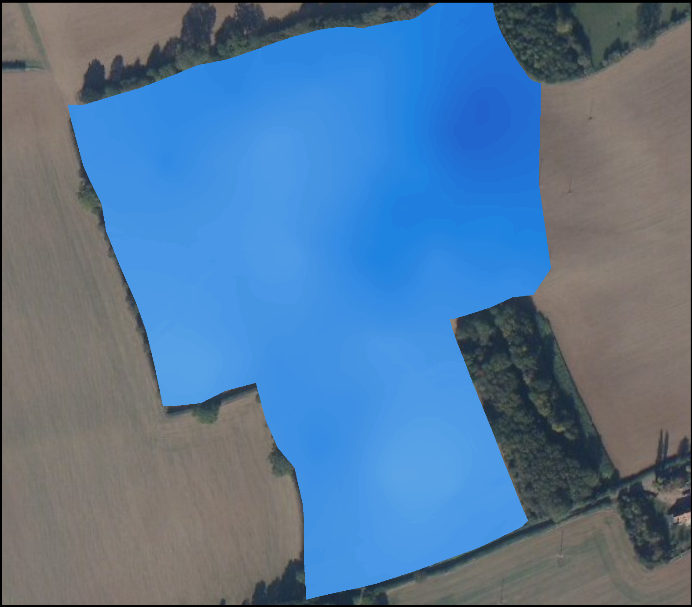  d |
| 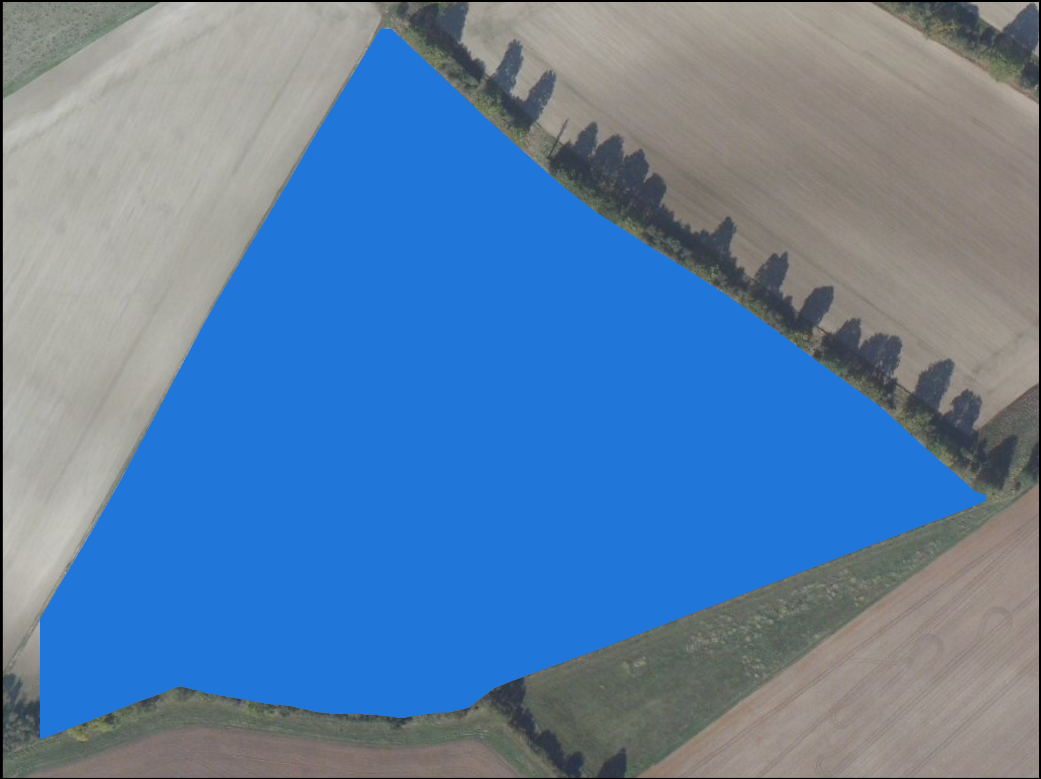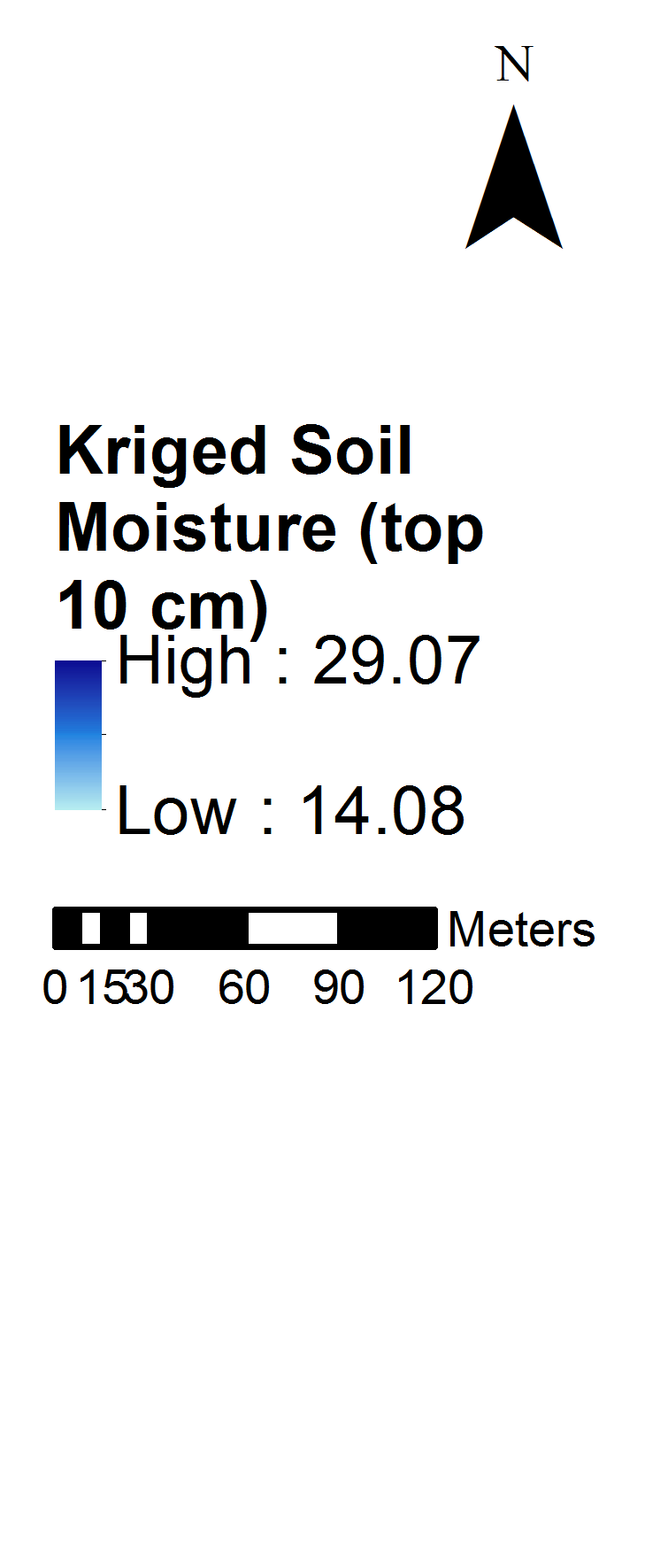  e | |

**Fig. S2** Maps showing the kriged soil organic matter measured by loss on ignition in each of the 5 fields a) Harpenden b) Redbourn, c) Haversham, d) Ivinghoe. No data was available for the field in Radbrook. The kriging was conducted using ordinary kriging based on the variogram fitted for that field.

| 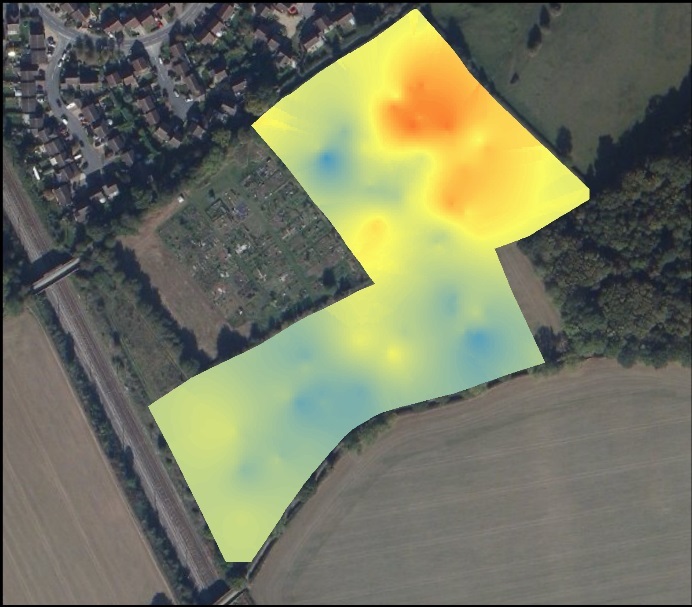  a | 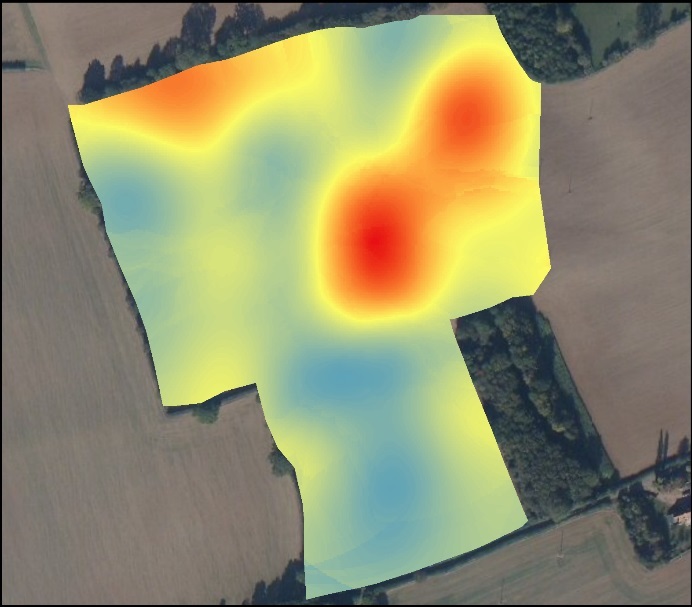  b | | |
| --- | --- | --- | --- |
| 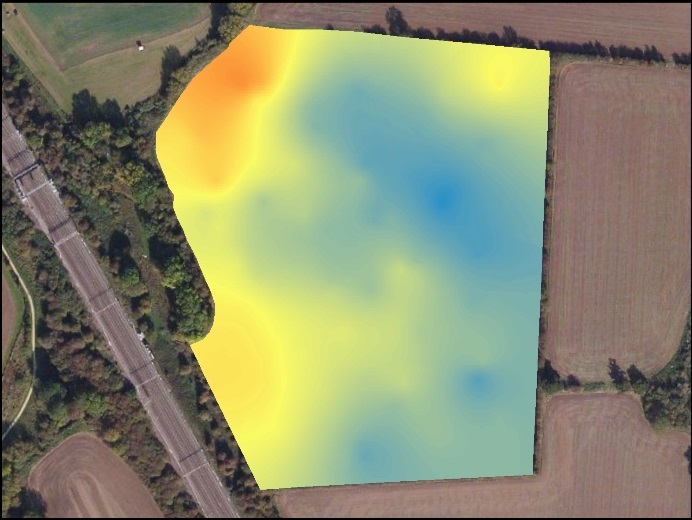  c  e | | 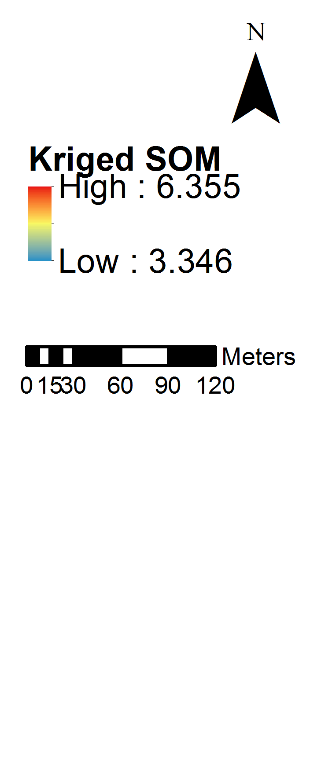 | |
| 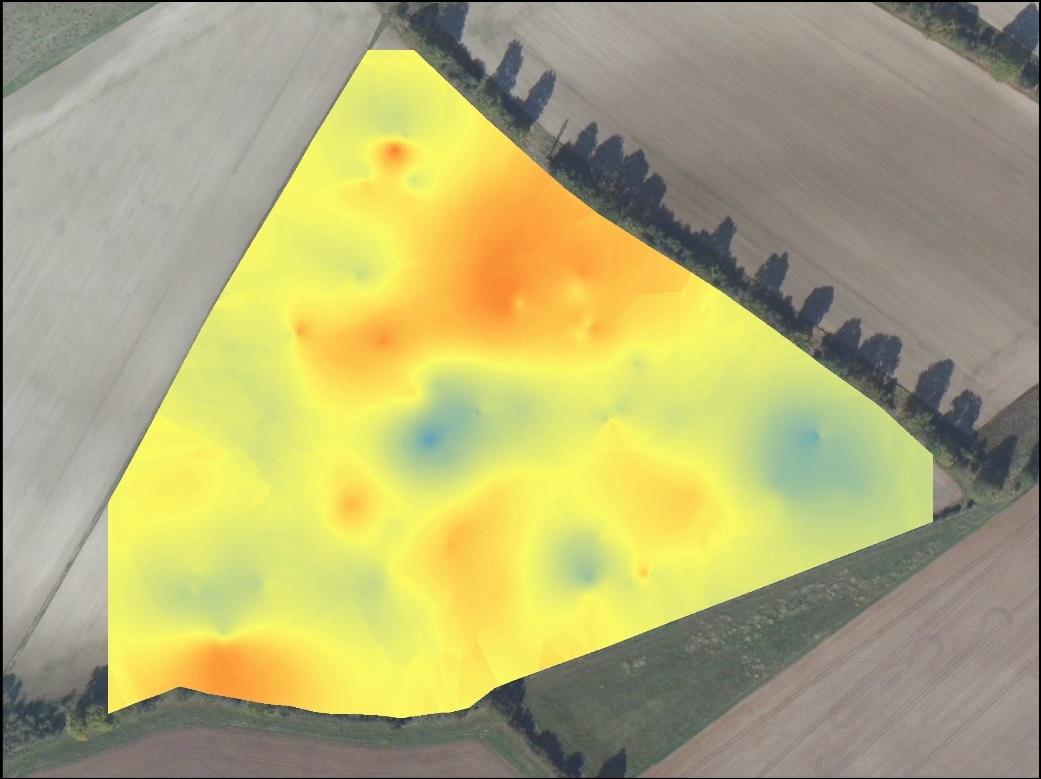  d | | |  |

**Fig. S3** Maps showing the kriged soil clay content in each of the 5 fields a) Radbrook b) Haversham, c) Harpenden, d) Redbourn, e) Ivinghoe, soil moisture is gravimetric in all cases except Radbrook where the volumetric moisture content is shown. The kriging was conducted using ordinary kriging based on the variogram fitted for that field.

e

| 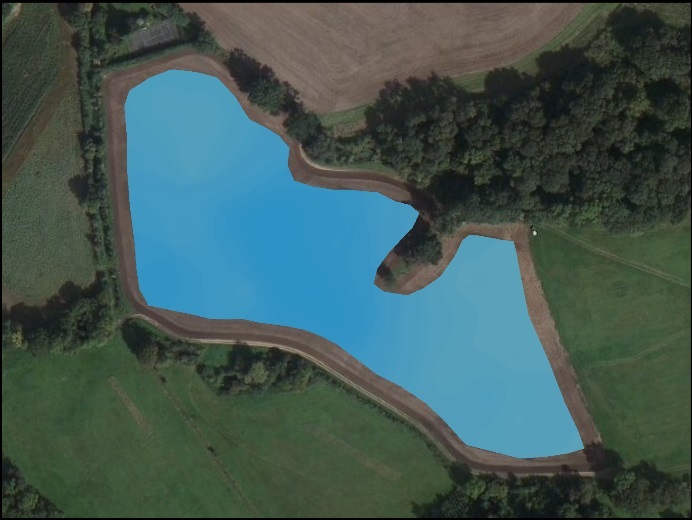  a | | 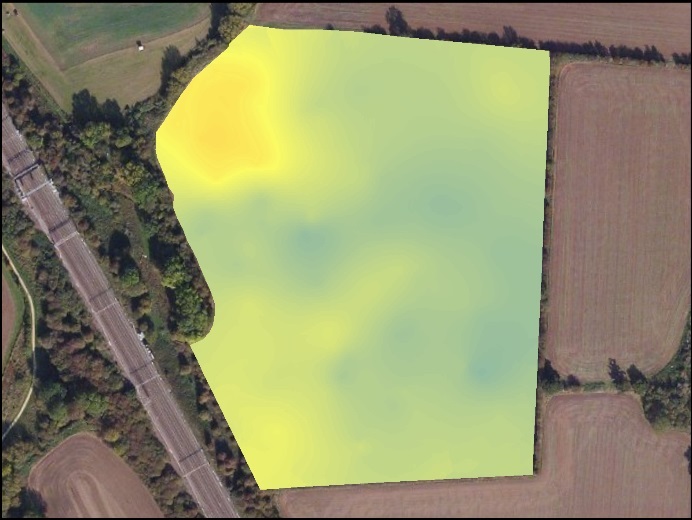  b |
| --- | --- | --- |
| 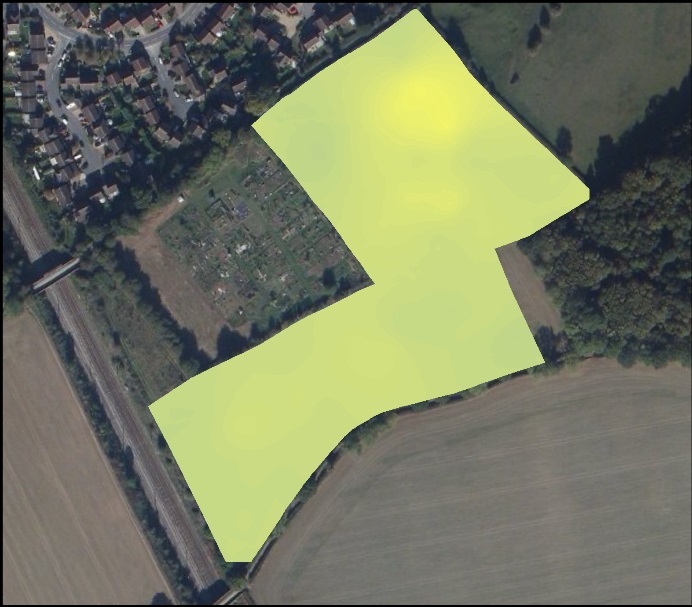  e  c | 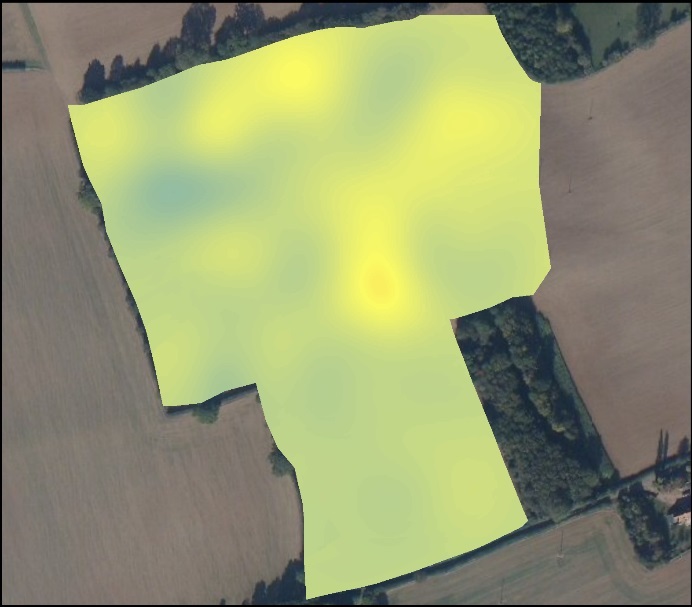  d | |
| 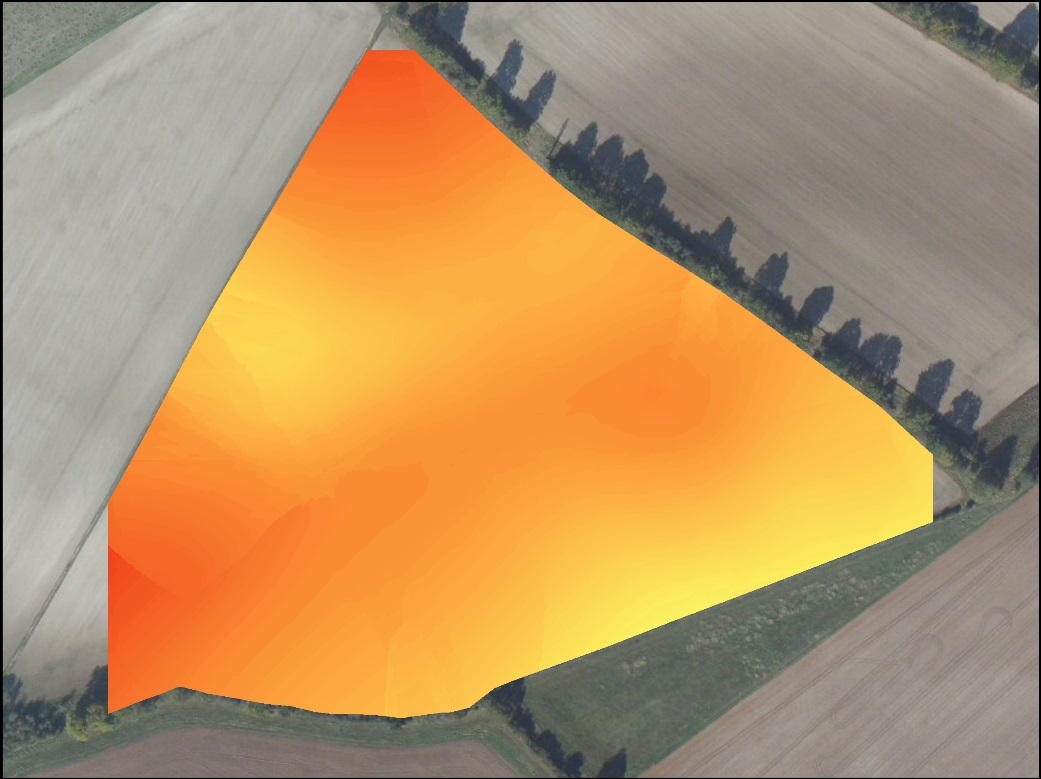 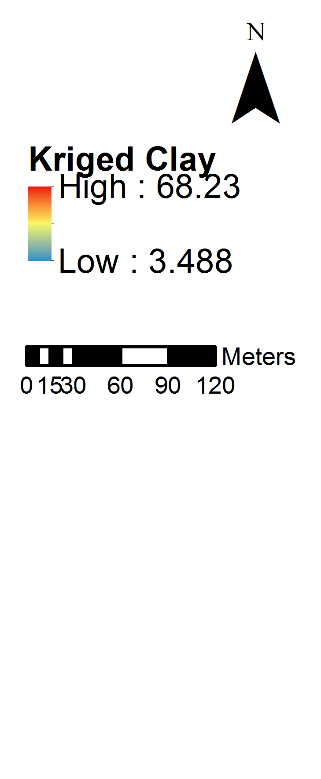 | | |

**Fig. S4** Maps showing the kriged soil pH in each of the 5 fields a) Radbrook b) Haversham, c) Harpenden, d) Redbourn, e) Ivinghoe, soil moisture is gravimetric in all cases except Radbrook where the volumetric moisture content is shown. The kriging was conducted using ordinary kriging based on the variogram fitted for that field.

a

| 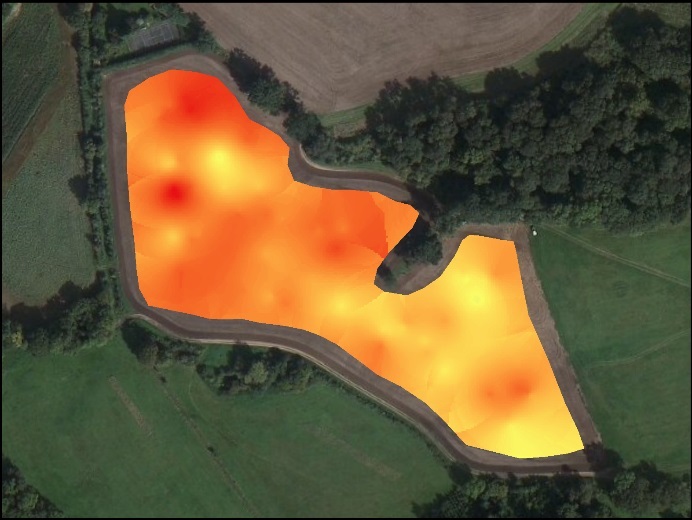 | | 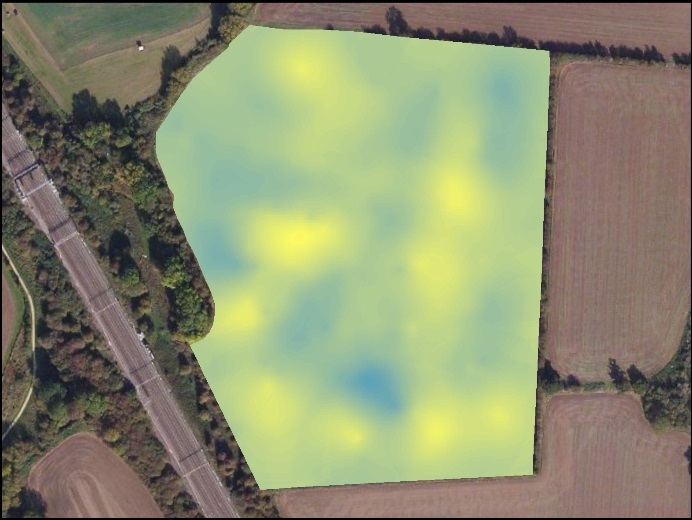  b |
| --- | --- | --- |
| 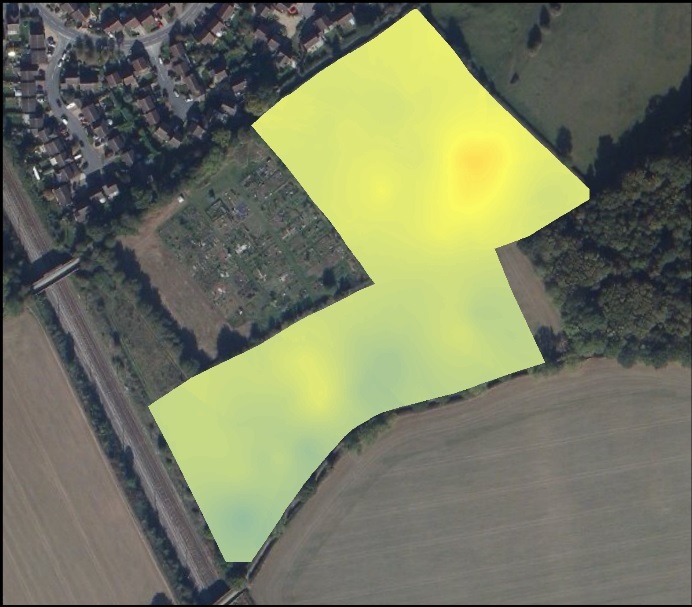  c  e | 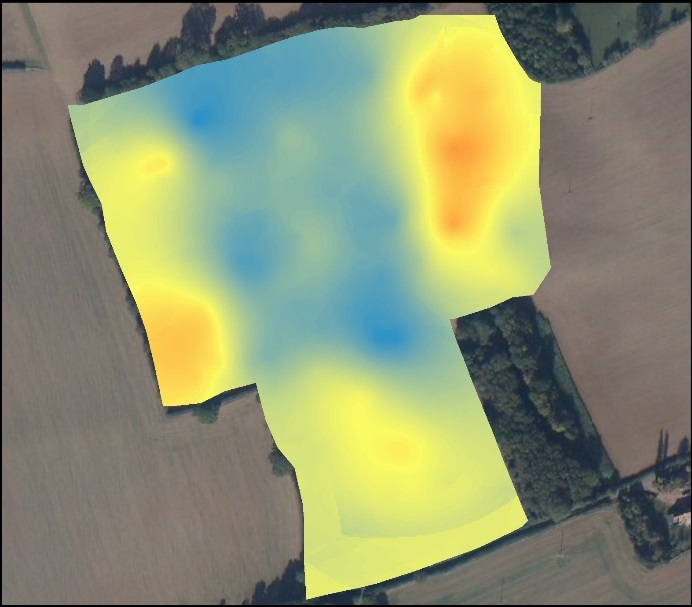  d | |
| 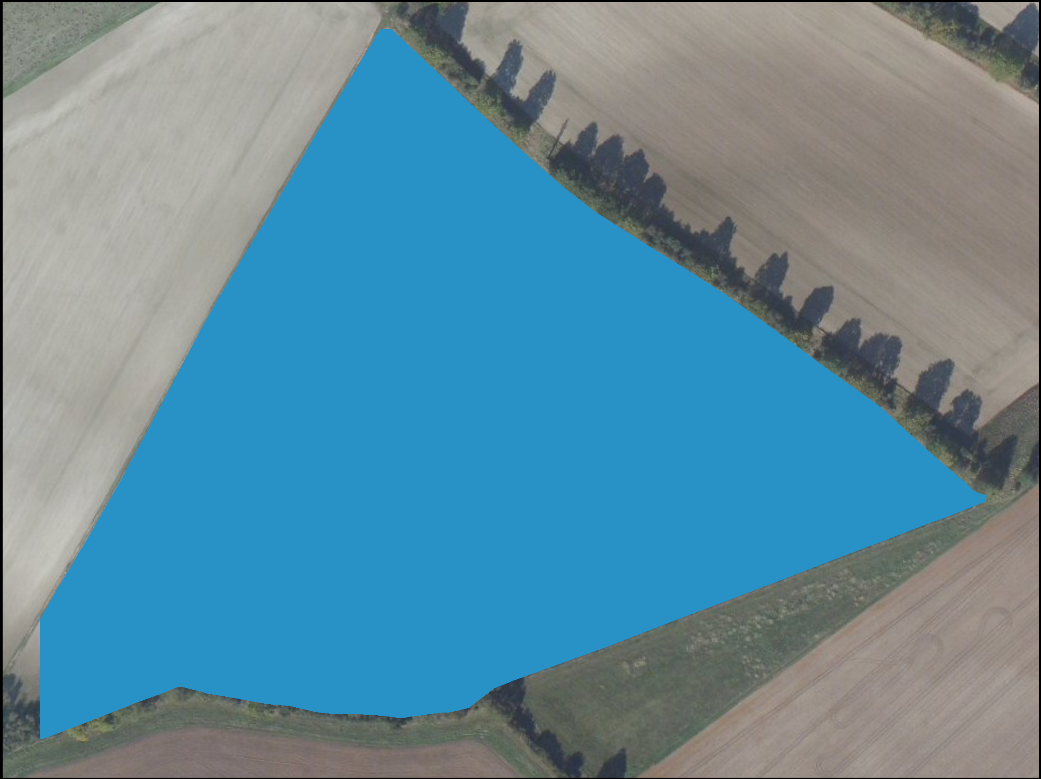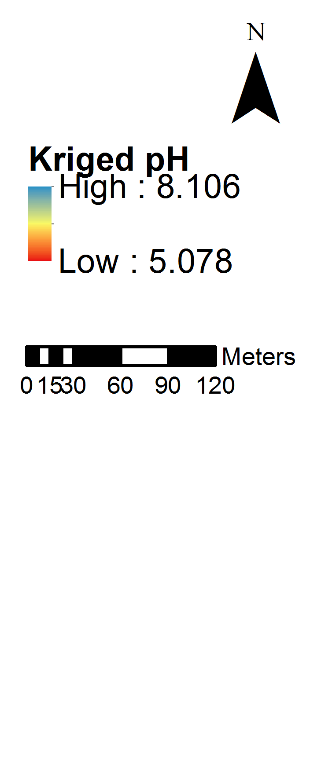  e | | |
